# Evaluation of triflumuron and pyriproxyfen as alternative candidates to control house fly, *Musca domestica* L. (Diptera: Muscidae), in Riyadh city, Saudi Arabia

**DOI:** 10.1371/journal.pone.0249496

**Published:** 2021-04-08

**Authors:** Saad M. Alzahrani

**Affiliations:** King Abdulaziz City for Science and Technology (KACST), Life Science and Environment Institute, Riyadh, Saudi Arabia; Al-Azhar University, EGYPT

## Abstract

This study was conducted to determine the susceptibility and resistance of some house fly strains of *Musca domestica* L. to the insect growth regulator insecticides triflumuron and pyriproxyfen in some locations in Riyadh city. Field-collected strains of *M*. *domestica* L. from five sites in Riyadh city that represented five slaughterhouse sites where flies spread significantly were tested against triflumuron and pyriproxyfen. Triflumuron LC_50_ values for the five collected strains ranged from 2.6 to 5.5 ppm, and the resistance factors (RFs) ranged from 13-fold to 27-fold that of the susceptible laboratory strain. Pyriproxyfen LC_50_ values for the field strains ranged from 0.9 to 1.8 ppm with RFs of 3-fold to 5-fold. These results indicate that pyriproxyfen is an effective insecticide to control house flies and should be used in rotation with other insecticides in the control programs applied by Riyadh municipality.

## Introduction

Globally, the house fly *Musca domestica* L. is considered a major domestic pest and a threat to public health, as this insect is the primary vector for a number of contagious disease agents, such as those causing cholera, typhoid, dysentery and some dermal diseases, in addition to trachoma and intestinal diseases [1–3]. Unsanitary conditions in sites such as slaughterhouses, dairies and livestock arenas provide optimal feeding and breeding environments for house flies, leading to disease transmission. Transmission can occur when insects make contact with human habitats [4]. Therefore, Riyadh municipality has put great effort into controlling house flies around the city.

In response to the intensive application of common used insecticides (such as pyrethroids, organophosphates and carbamates) in the region to control *M*. *domestica* L. these insects eventually developed a strong resistance toward these chemicals in Saudi Arabia [5–7].

Insect growth regulators (IGRs) are promising candidates for controlling house flies either by rotating them with other insecticides from different chemical groups (IRAC classification) [8], or with other control approaches (e.g., biological and physical controls). This class of insecticides is distinguished by their interference with the developmental processes in insects, which make them remarkably harmless for the environment [9–11].

The IGRs cyromazine and pyriproxyfen were very toxic to *Hermetia illucens* L. (Diptera, Stratiomyidae) flies after the larvae were fed an IGR-treated diet. Death occurred before larvae reached the prepupal stage, with low LC_50_ values of 0.13 and 0.25 ppm for cyromazine and pyriproxyfen, respectively [12]. Another IGR, diflubenzuron, was effective in controlling house flies, and elimination of adult emergence occurred after eggs were treated at a concentration of 250 ppm [13].

Pyriproxyfen can also cause failed adult emergence from the pupal stage due to the damage and deformities suffered during the larval stage. Larvae of *Hyposoter didymator* treated with 75, 500 and 1000 ppm pyriproxyfen failed to emerge from their host [14]. In house flies, triflumuron and pyriproxyfen reduced adult emergence by 98% after 1^st^ instar larvae were exposed to 0.5 ppm of both IGRs [15]. A laboratory study showed the larvicidal effect of triflumuron by oral application to the 2^nd^ instar larvae of *M*. *domestica* L., and the IGR had a significant effect on larval mortality compared to that in untreated insects [16]. In Pakistan, six field-collected strains of *M*. *domestica* L. exhibited low to moderate levels of resistance against five IGRs, including triflumuron and pyriproxyfen. The resistance factor (RF) ranged from 1.45-fold to 3.68-fold for triflumuron and 4.13-fold to 11.63-fold for pyriproxyfen, demonstrating IGRs as effective insecticides to control house flies if resistance management practices are followed [17].

This study was designed to test and evaluate the toxicity of two IGRs, triflumuron and pyriproxyfen, which are herein recommended for house fly control programs in Riyadh city, Saudi Arabia.

## Materials and methods

### Chemicals

The insect growth regulators used in this study were pyriproxyfen 0.5 WDG (Sumilarv^®^, Sumitomo Chemical Co., Ltd), which was supplied by Agricultural office T.C. The second was triflumuron, 25 WP (Baycidal^®^, Bayer AG), which was purchased from Quraish T.C., Riyadh, Saudi Arabia.

### Insect strains

#### Laboratory strain

Adult flies, *M*. *domestica* L., were collected by sweep net from a sheep pen located in Alhasa oasis, eastern Saudi Arabia (25°22’41.4"N 49°37’05.9"E). The collected insects were transferred to the laboratory and kept in wooden cages (60 x 60 x 60 cm) wrapped with metal wire with a hole width of 1 mm. The insects were reared with standard nutrition [18–20], temperature (25±3°C), relative humidity (30–40%) and a photoperiod (12:12 hours) conditions, without any exposure to pesticides.

Adult flies were fed a mixture of powdered milk, sugar and yeast. The weight ratio of the mixture was 2:1:1 in 100 mL water. Cotton soaked in a 10% sugar solution was also provided as a source of energy for adult insects. ~40 g of the mixtures were placed in 9-cm petri dishes and changed every 2–3 days. For larval feed, a mixture of pigeon manure (as an organic matter) and bran was applied in addition to a 10 mL of 5% ammonium solution to attract females for egg laying, with a weight ratio of 3:1 for manure and bran. Similarly, ~40 g of the mixtures were placed in 9-cm petri dishes [18–20].

Rearing and conservation were carried out for 30 generations to obtain a colony of house flies that contained a range of genetic backgrounds (resistant, tolerant, and susceptible), and their susceptibility was tested during the breeding period until they reached the susceptibility levels required for investigation.

#### Field strains

The field strains of the house fly *M*. *domestica* L. were collected from five slaughterhouses in Riyadh city that were highly infested with house flies. These slaughterhouses rely upon the Riyadh municipality authority and monitoring which includes house fly control. The sample collection was authorized by the municipality after coordinating with the slaughterhouses’ managements. These slaughterhouses are Northside slaughterhouse (24°45’23.4"N 46°39’59.6"E), Aziziya slaughterhouse (south) (24°35’05.7"N 46°44’47.7"E), As Saadah district slaughterhouse (east) (24°42’30.2"N 46°51’17.1"E), West Riyadh slaughterhouse (24°34’12.1"N 46°30’27.3"E) and Al-Munsiyah slaughterhouse (northeast) (24°49’40.5"N 46°47’46.5"E). These sites represent the main four directions of the city (north, east, west and south), and the distance between any two sites was not less than 3.22 km (2 mi) to ensure that the collected insects from the sites did not overlap [21]. Insects were collected using ovipositional media, containing 5 kg of sheep manure mixed with 1 kg of bran in addition to 100 mL of 5% ammonium solution. The trays containing the mixture were placed in sites next to waste containers for a period of 5–6 days during which the humidity was increased in the media once or twice according to air temperature and relative humidity. After that, the trays were returned to the laboratory, where ~50 of third-instar larvae were immediately isolated for IGRs bioassay.

### Insecticide bioassay

The 3^rd^ instar larvae were treated by feeding according to the method indicated by [15]. Ten larvae were placed in a specimen cup containing a mixture of 5 grams of mouse chow with 5 mL of IGR solution, and water was used for the control. Each test concentration of IGR was repeated three times, and the range of concentrations was 0, 0.156, 0.312, 0.625, 1.25, 2.5, 5, 10 and 20 ppm. Cumulative mortality was calculated after 14 days of treatment, as pupae that failed to emerge were considered dead. Each experiment was repeated twice with three technical replicates per insecticide concentration.

Probit analysis [22] was carried out using software (LdP Line, copyright 2000 by Ehab Mostafa Bakr, Cairo, Egypt) to compute the lethal median concentration (LC_50_), and the 95% confidence intervals. Mortality was corrected by the Abbot formula [23]. One way ANOVA followed by Dunnett’s multiple comparisons was carried out for comparing the significance of differences between the field strains and the laboratory one. SigmaPlot was employed to generate graphs.

The resistance factor (RF) was calculated according to the equation:

$$\frac{{LC}_{50}\;for\;tested\;strain}{{LC}_{50}\;for\;laboratory\;strain}$$

The RF determines the increase in resistance in the tested strains compared to that in the laboratory strain. If the resistance factor is ≥10-fold that of the laboratory strain, the strain is designated as resistant [24].

## Results

**Fig 1:**
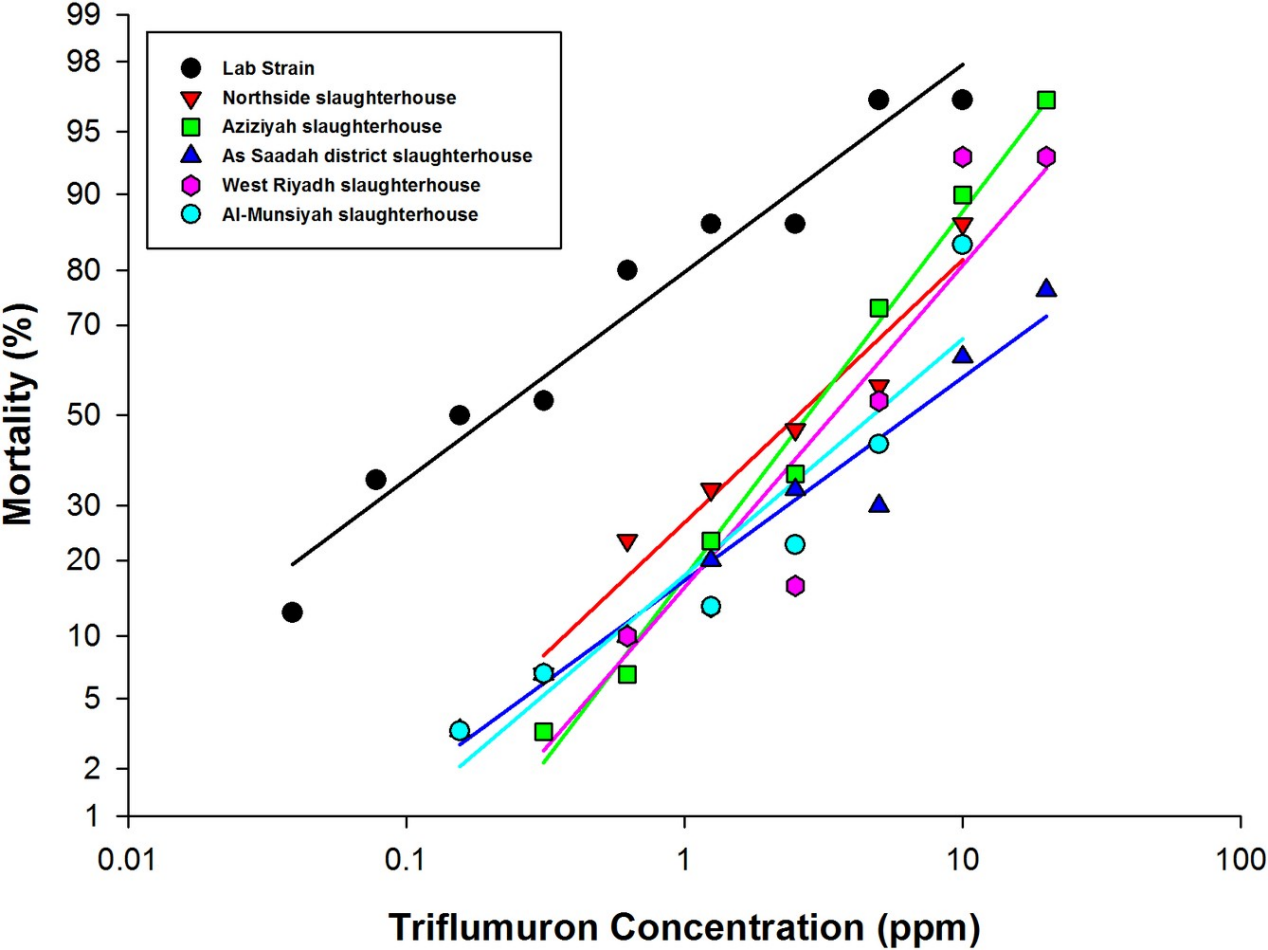
Mortality induced by exposing 3^rd^ instar larvae of *M*. *domestica* L. to triflumuron. Larvae were fed different IGR concentrations, and the cumulative mortality was calculated after 14 days of exposure.

**Fig 2:**
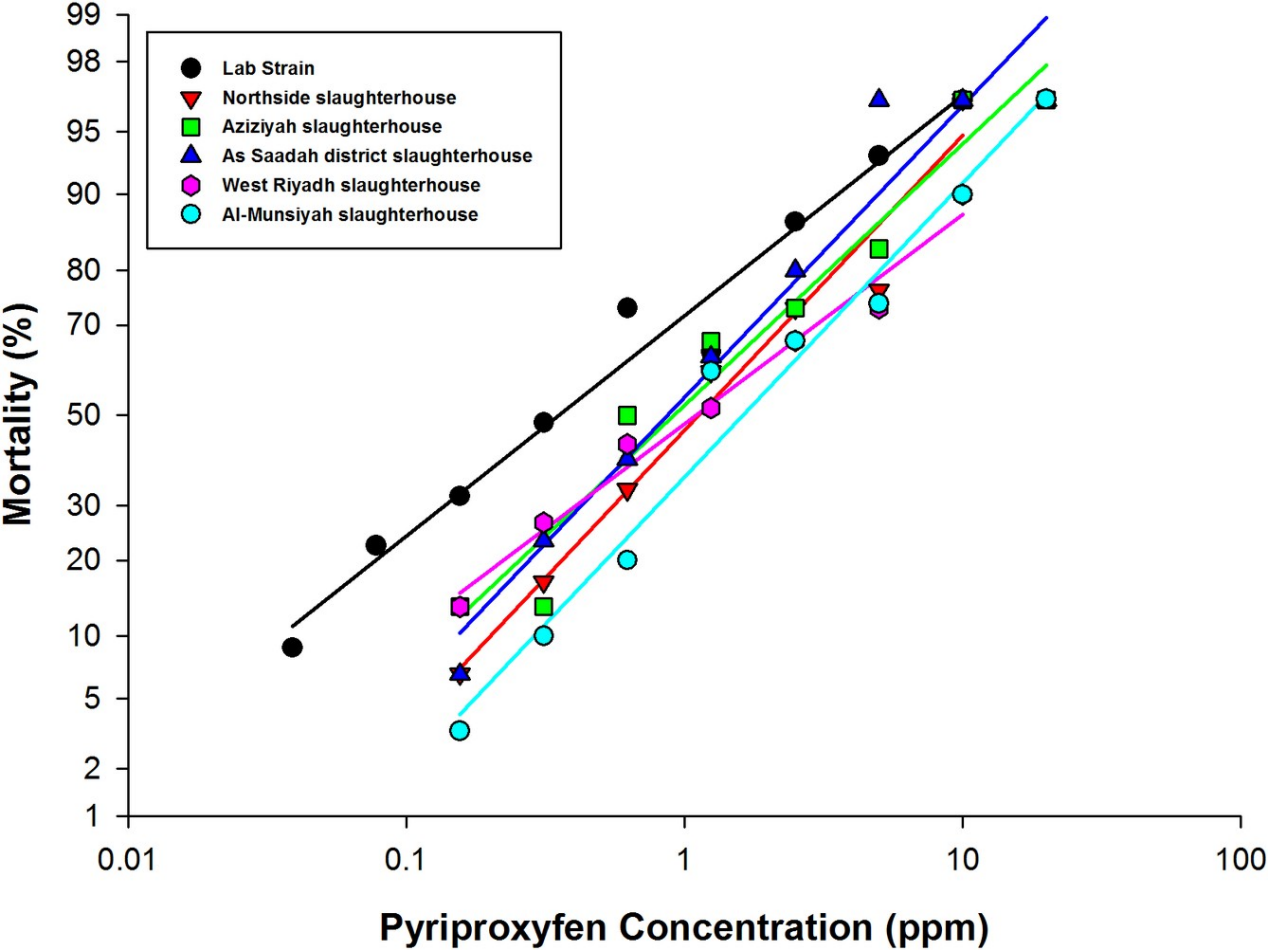
Mortality induced by exposing 3^rd^ instar larvae of *M*. *domestica* L. to pyriproxyfen. Larvae were fed different IGR concentrations, and the cumulative mortality was calculated after 14 days of exposure.

All fly strains exhibited concentration-dependent mortality to both IGRs (Table 1, Figs 1 and 2). The triflumuron LC_50_ for the laboratory strain was 0.20 (0.16–0.24) ppm, while for pyriproxyfen the LC_50_ was 0.35 (0.29–0.42) ppm. The response to both IGRs varied among the field-collected strains. All of these strains were resistant to the IGR triflumuron with an RF > 10-fold. The triflumuron LC_50_ for the Northside slaughterhouse strain was 2.62 (2.20–3.17) ppm, and that for the Aziziyah slaughterhouse strain was 2.86 (2.52–3.25) ppm. The strains collected from the As Saadah district slaughterhouse, West Riyadh slaughterhouse and Al-Munsiyah slaughterhouse displayed higher resistance to the IGR with LC_50_ values of 5.48 (4.40–7.02), 4.62 (2.65–11.91) and 4.72 (3.23–8.58), respectively (Table 1, Fig 1).

**Table 1:** Triflumuron and pyriproxyfen LC_50_ values and resistance factors for *M*. *domestica* L. strains.

IGR	Strain	LC_50_ (CI.)	LC_95_ (CI.)	Slope±SE	R	*X*^*2*^	*df*	RF
Triflumuron	Laboratory strain	0.20 (0.16–0.24)	3.97 (2.81–6.14)	1.26±0.09	0.98	12.51	8	
	Northside slaughterhouse	2.62 (2.20–3.17)**	35.14 (22.89–62.84)	1.46±0.12	0.98	9.08	6	13.1
	Aziziyah slaughterhouse	2.86 (2.52–3.25)***	16.23 (12.97–21.37)	2.18±0.13	0.99	5.08	7	14.3
	As Saadah district slaughterhouse	5.48 (4.40–7.02)****	110.95 (67.48–211.63)	1.26±0.09	0.99	0.76	7	27.4
	West Riyadh slaughterhouse	4.62 (2.65–11.91)****	41.78 (42.94–363.88)	1.72±0.13	0.94	27.81	6	23.1
	Al-Munsiyah slaughterhouse	4.72 (3.23–8.58)****	35.26 (27.77–146.78)	1.88±0.15	0.97	15.46	6	23.6
Pyriproxyfen	Laboratory strain	0.35 (0.29–0.42)	7.09 (5.10–10.63)	1.26±0.07	0.99	13.11	9	
	Northside slaughterhouse	1.19 (1.01–1.41)****	14.21 (10.15–21.87)	1.53±0.11	0.99	8.32	7	3.4
	Aziziyah slaughterhouse	0.91 (0.47–1.44)***	11.38 (8.70–47.41)	1.50±0.13	0.99	15.06	6	2.6
	As Saadah district slaughterhouse	0.86 (0.74–1.00)**	6.88 (5.09–10.17)	1.82±0.13	0.99	1.24	6	2.5
	West Riyadh slaughterhouse	1.10 (0.90–1.34)****	26.50 (16.91–48.15)	1.19±0.09	0.99	3.60	7	3.1
	Al-Munsiyah slaughterhouse	1.76 (1.50–2.07)****	15.96 (12.23–22.03)	1.72±0.10	0.99	2.88	7	5.0

*p < 0.*05*, **p < 0.*005*, ***p < 0.001 and ****p < 0.*0001*.

A comparison with one way ANOVA followed by Dunnett’s multiple test was performed to identify significant differences in LC_50_ values due to IGR exposure comparing to the laboratory strain.

Pyriproxyfen was relatively more toxic to the field-collected flies. The RF of all strains was < 10-fold. The LC_50_ values for the Northside slaughterhouse strain, Aziziyah slaughterhouse strain, As Saadah district slaughterhouse strain, West Riyadh slaughterhouse strain and Al-Munsiyah slaughterhouse strain were 1.19 (1.01–1.41), 0.91 (0.47–1.44), 0.86 (0.74–1.00), 1.10 (0.90–1.34) and 1.76 (1.50–2.07) ppm, respectively (Table 1, Fig 2).

**Fig 3:**
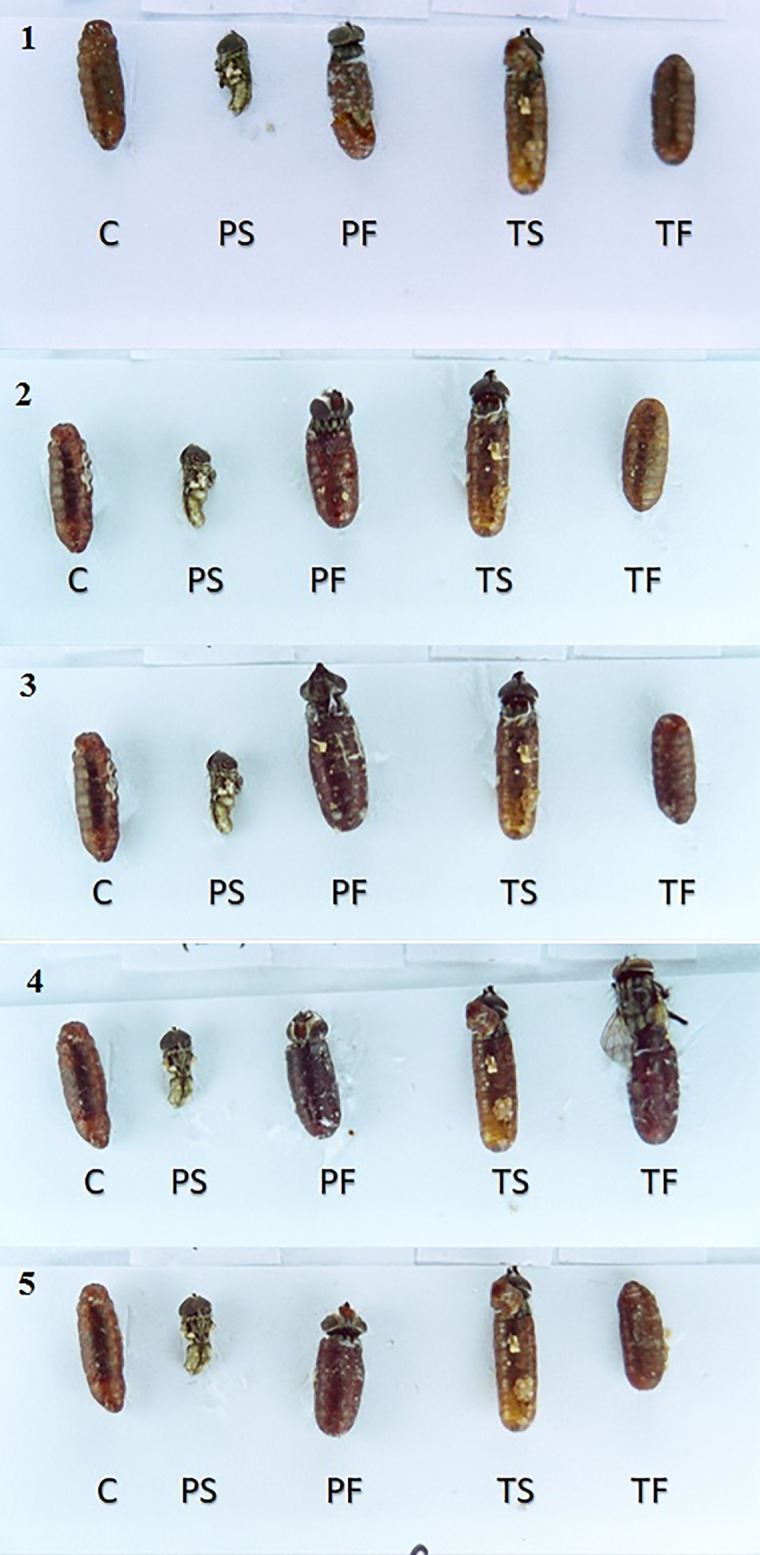
Pupae deformity induced by pyriproxyfen and triflumuron on house fly strains. Insects failed to emerge from the pupal stage after suffering deformations from exposing 3^rd^ instar larvae to 0.625 ppm from each IGR. 1. Northside slaughterhouse strain, 2. Aziziya slaughterhouse strain, 3. As Saadah district slaughterhouse strain, 4. West Riyadh slaughterhouse strain and 5. Al-Munsiyah slaughterhouse strain. C: Control, PS: pyriproxyfen susceptible-strain, PF: Pyriproxyfen field-strain, TS: triflumuron susceptible-strain, TF: triflumuron field-strain.

**Fig 4:**
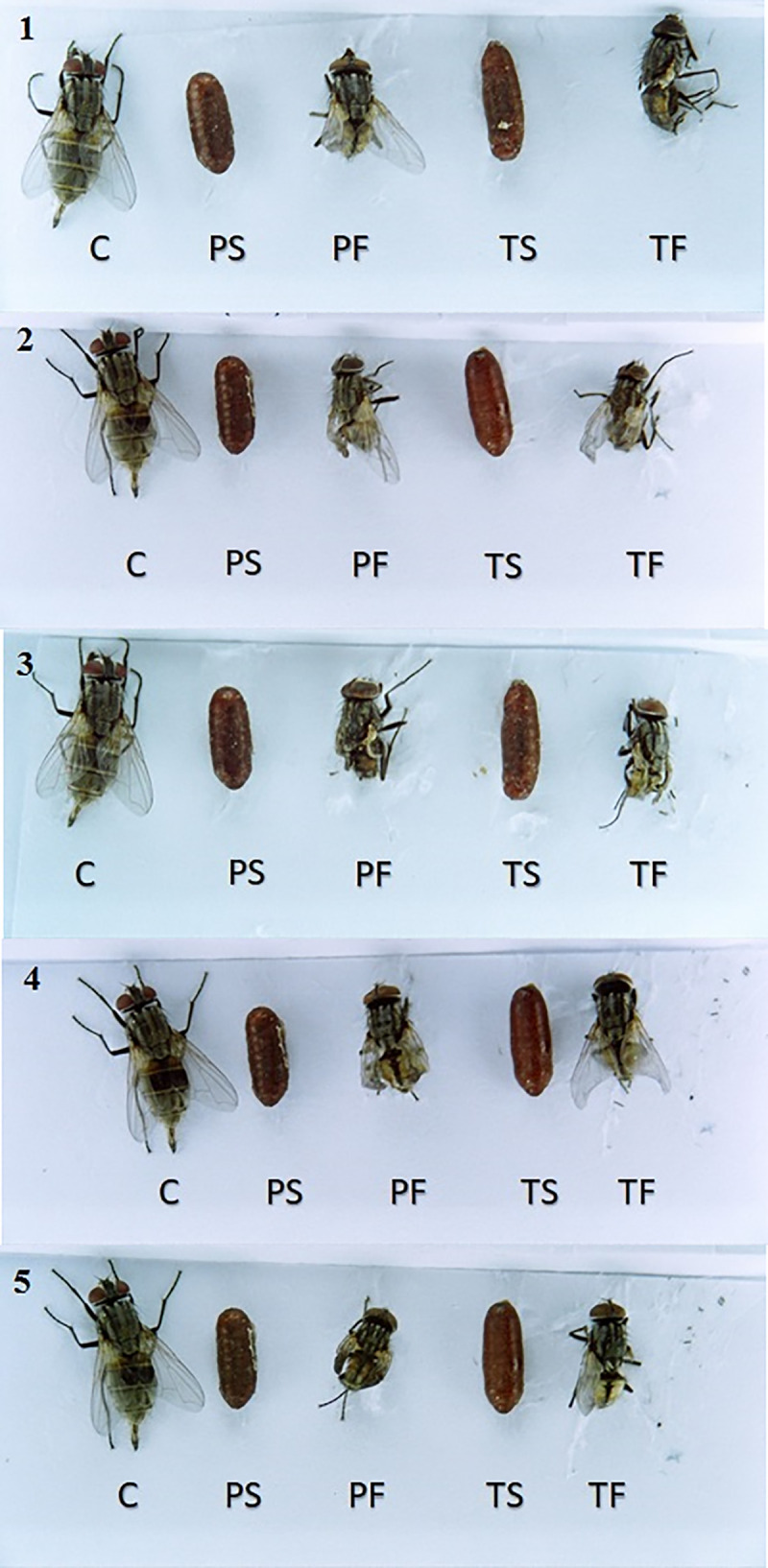
Adult deformity induced by pyriproxyfen and triflumuron on house fly strains. Adult insects deformation is observable after exposing 3^rd^ instar larvae to 0.625 ppm from each IGR. 1. Northside slaughterhouse strain, 2. Aziziya slaughterhouse strain, 3. As Saadah district slaughterhouse strain, 4. West Riyadh slaughterhouse strain and 5. Al-Munsiyah slaughterhouse strain. C: Control, PS: pyriproxyfen susceptible-strain, PF: Pyriproxyfen field-strain, TS: triflumuron susceptible-strain, TF: triflumuron field-strain.

Another insecticidal effect of pyriproxyfen and triflumuron is the ability to cause abnormalities in adult flies after emergence. All field-collected strains exhibited deformities in adult insects and pupae at a concentration of 0.625 ppm (Tables 2 and 3, Figs 3 and 4). Table 3 depicts the number of deformities resulting from pyriproxyfen at each concentration in each strain. From the results, it is clear that the strains of the Northside slaughterhouse, West Riyadh slaughterhouse and Al-Munsiyah slaughterhouse showed a number of abnormalities at lower concentrations (0.625, 1.25 and 2.5 ppm) (Table 3), while direct death (failure to emerge from the pupal stage) was observed at higher concentrations (5, 10 and 20 ppm) (Table 3). This may mean that these strains have begun to develop resistance against pyriproxyfen, as flies were able to complete the pupal stage and emerge as adult insects at lower concentrations. At higher concentrations, death occurred during the pupal stage, and adult flies failed to emerge.

**Table 2:** Number of deformity cases induced by triflumuron at each concentration.

Conc. (ppm) Strain	0.625		1.25		2.5		5		10		20	
	Def.*	Death	Def. *	Death	Def.*	Death	Def.*	Death	Def.*	Death	Def.*	Death
Northside slaughterhouse	1	6	5	5	8	6	0	17	1	25	0	30
Aziziyah slaughterhouse	0	3	0	8	2	10	0	23	0	28	0	30
As Saadah district slaughterhouse	0	4	6	1	2	9	6	4	2	18	0	24
West Riyadh slaughterhouse	3	0	4	0	5	0	5	8	2	26	2	26
Al-Munsiyah slaughterhouse	0	0	0	4	7	0	4	9	2	24	0	30
Total	4	13	15	18	24	25	15	61	7	121	2	140

*Deformation.

Thirty, third instar larvae of M. domestica L. were exposed to a range of triflumuron concentrations.

**Table 3:** Number of deformity cases induced by pyriproxyfen at each concentration.

Conc. (ppm) Strain	0.625		1.25		2.5		5		10		20	
	Def.*	Death	Def.*	Death	Def.*	Death	Def.*	Death	Def.*	Death	Def.*	Death
Northside slaughterhouse	2	8	4	14	6	16	2	21	0	29	0	30
Aziziyah slaughterhouse	6	10	2	19	0	23	0	26	0	30	0	30
As Saadah district slaughterhouse	3	10	2	18	3	22	0	30	0	30	0	30
West Riyadh slaughterhouse	5	8	5	11	4	16	3	19	3	24	1	29
Al-Munsiyah slaughterhouse	6	0	6	12	7	13	4	19	1	26	1	28
Total	22	36	19	74	20	90	9	115	4	139	2	148

*Deformation.

Thirty, third instar larvae of M. domestica L. were exposed to a range of pyriproxyfen concentrations.

With triflumuron treatment, no abnormalities occurred at lower concentrations, with the exception of three individuals of the Aziziyah slaughterhouse strain and one individual of the Northside slaughterhouse strain (Table 2). However, at higher concentrations (1.25, 2.5 and 5 ppm), deformation occurred. At even higher concentrations (10 and 20 ppm), direct death was observed as adult flies failed to emerge from pupae (Table 2, Figs 3 and 4).

## Discussion

This study was conducted to determine the susceptibility and resistance of field-collected strains of the house fly *M*. *domestica* L. in Riyadh city to two insect growth regulators, triflumuron and pyriproxyfen. The exhaustive usage of conventional insecticides such as organophosphates, pyrethroids and carbamates by Riyadh municipality for controlling house flies has led to the emergence of strong resistance among insect populations toward these insecticides, decreasing their efficacy [6, 7]. Moreover, the ability of insects, especially house flies such as *M*. *domestica* L., to develop resistance against different groups of conventional insecticides has suggested the exploration of the toxicity of these two IGRs, which this study evaluated as candidates for house fly control.

The insecticidal activity of IGRs interferes with biological pathways or processes that are crucial for insect growth and development. The effects can occur either by inhibiting chitin synthesis during the development process or by simulating the juvenile hormone, which leads to disorganization of the required periods for insect growth during each stage [10, 25].

The resistance levels toward conventional insecticides in house fly populations in Riyadh city [6, 7] have increased control costs, with the need for higher concentrations, which in turn will pose environmental threats. The results of this study illustrate a promising candidate to be utilized in house fly control programs. The field populations of insects in Riyadh demonstrated susceptibility to the IGR pyriproxyfen compared with the laboratory strain, with resistance factor values < 10 in all tested strains (Table 1).

This result is consistent with the findings of researchers [13] who treated *M*. *domestica* L. with pyriproxyfen. The mortality rates were 87.2% on average for the SRS/WHO susceptible strain and 84.3% for the REKe field strain, which indicates that there is no resistance to the insecticide and consequently it has high toxicity against field-collected house flies. Moreover, Malaysian scientists [15] demonstrated the high effectiveness of pyriproxyfen and triflumuron in inhibiting the development of adult house flies sampled from dairy farms. Additionally, in the case of pyriproxyfen, the findings of this study coincide with what Khan et al. reported from Pakistan [17]. The findings showed the efficiency and importance of utilizing IGRs, including pyriproxyfen, in house fly control programs, wherein these chemicals were toxic against field-collected strains.

All experimental strains used in this study showed susceptibility to the IGR pyriproxyfen; on the other hand, all of them were resistant to the other IGR, triflumuron. This indicates that all field-collected strains were able to resist triflumuron either from the previous exposure to the IGR or cross-resistance with other insecticides applied to control house flies by Riyadh municipality. This finding supports the claims that these insects have the ability to develop resistance against different types of insecticides. This could be due to the introduction of triflumuron by Riyadh municipality to the intensive control campaign to control disease vectors including house flies and mosquitoes, which targeted mainly waste containers in slaughterhouses, restaurants, vegetable and meat markets and livestock markets as breeding sites for *M*. *domestica* L. [26].

Table 3 and Figs 3 and 4 show the number of cases of abnormalities for each concentration of pyriproxyfen in relation to the sites. Failure to emerge from the pupal stage only at higher concentrations (5 ‒ 10 ‒ 20 ppm) may mean that these strains are at the early stage of developing resistance against pyriproxyfen, as the insects were able to complete the pupal phase and emerge to the adult phase at low concentrations despite suffering deformations, compared to direct mortality at high concentrations. For triflumuron, Table 2 and Figs 3 and 4 indicate the number of deformations for each IGR concentration at all sites except for the Aziziyah slaughterhouse, showing that the strains had deformations at medium concentrations (1.25 ‒ 2.5 ‒ 5 ppm). While at the lower concentration (0.625 ppm) deformation was not observed except for three cases in the West Riyadh slaughterhouse strain and a case in the Northside slaughterhouse strain. At higher concentrations only (10 ‒ 20 ppm), direct death (failure to emerge from the pupal phase) occurred, which may have been due to the emergence of resistance in the strains against triflumuron or cross-resistance as a result of the Riyadh municipality’s usage of insecticides from the same group.

## Conclusion and recommendations

The IGR pyriproxyfen showed efficacy against the tested strains; therefore, it is recommended that it be included in house fly control programs. The results of this study show the need to periodically conduct susceptibility assessments to determine the level of resistance in house flies against insecticides used in the control programs.

House fly control agencies and authorities should reassess the routine spraying of insecticides, which can lead to the development of resistance in domestic populations of insects. In addition, the development of a new program is recommended in which the use of insecticides from various chemical groups is recycled to break the phenomenon of resistance development or at least to reduce the speed of the emergence of these resistant strains.

## Supporting information

S1 File Read me. (DOCX)

S2 File Dataset. (ZIP)

S3 File LdP line files. (ZIP)

S4 File SigmaPlot files. (ZIP)

S5 File GraphPad prism files. (ZIP)
