# Supplementary material for: Evaluation of triflumuron and pyriproxyfen as alternative candidates to control house fly, Musca domestica L. (Diptera: Muscidae), in Riyadh city, Saudi Arabia
Source: PLoS One. 2021 Apr 8;16(4):e0249496. doi: 10.1371/journal.pone.0249496 (PMC8031380; doi:10.1371/journal.pone.0249496)
Supplement: S1 File — (DOCX) [file pone.0249496.s001.docx]

**Evaluation of Triflumuron and Pyriproxyfen as Alternative Candidates to Control House Fly, *Musca domestica* L. (Diptera: Muscidae), in Riyadh City, Saudi Arabia**

Saad M. Alzahrani (smzahrni@kacst.edu.sa)

Here I highlight the software that has been utilized in this study. Each software serves a specific purpose as follow:

- Microsoft Excel: scoring averaging mortality percentage
- LdP Line (<http://www.ehabsoft.com/ldpline/>): conducting probit analysis to compute the LC_50s_


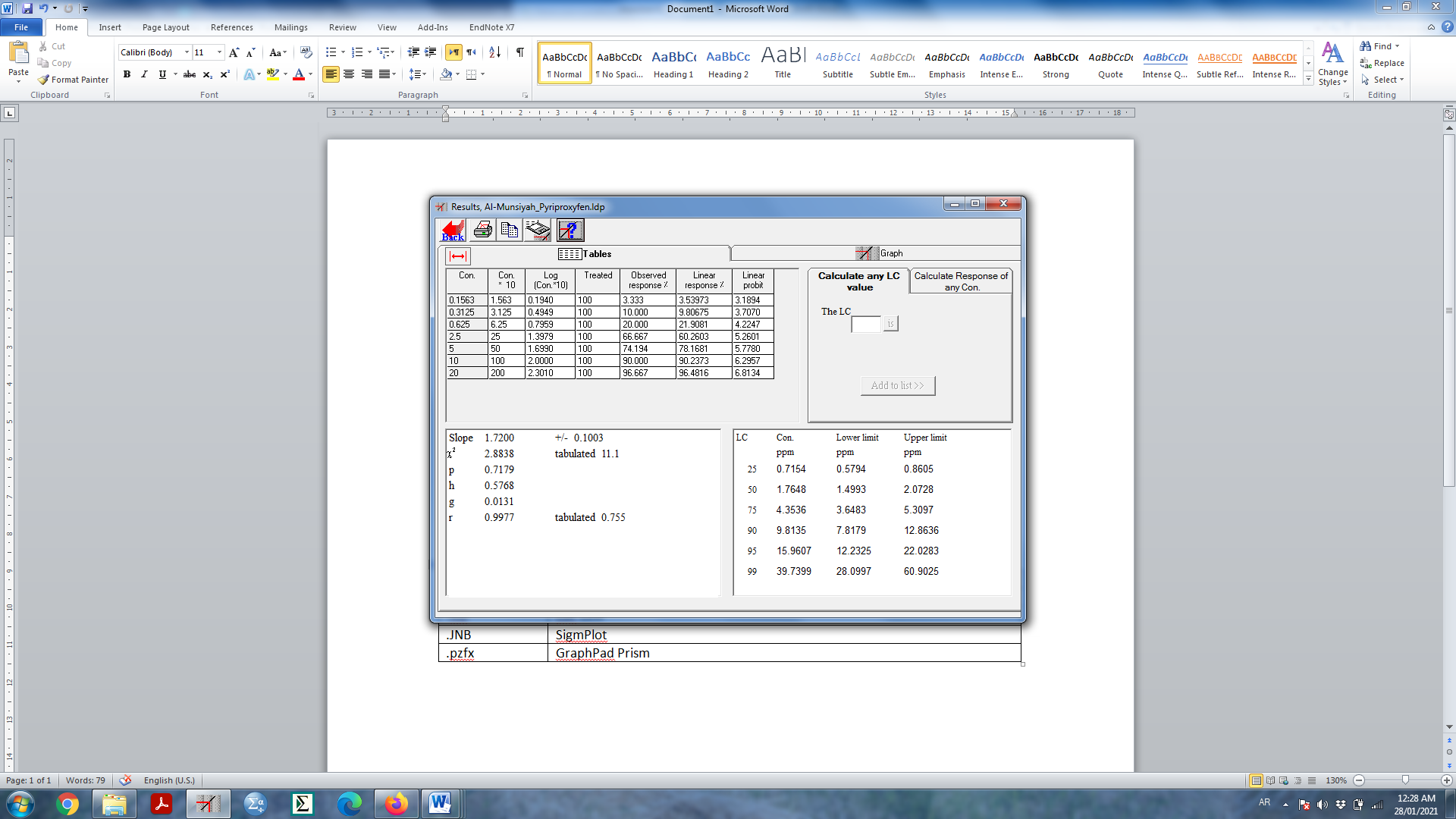


- SigmPlot (<https://systatsoftware.com/products/sigmaplot/>): generating graphs


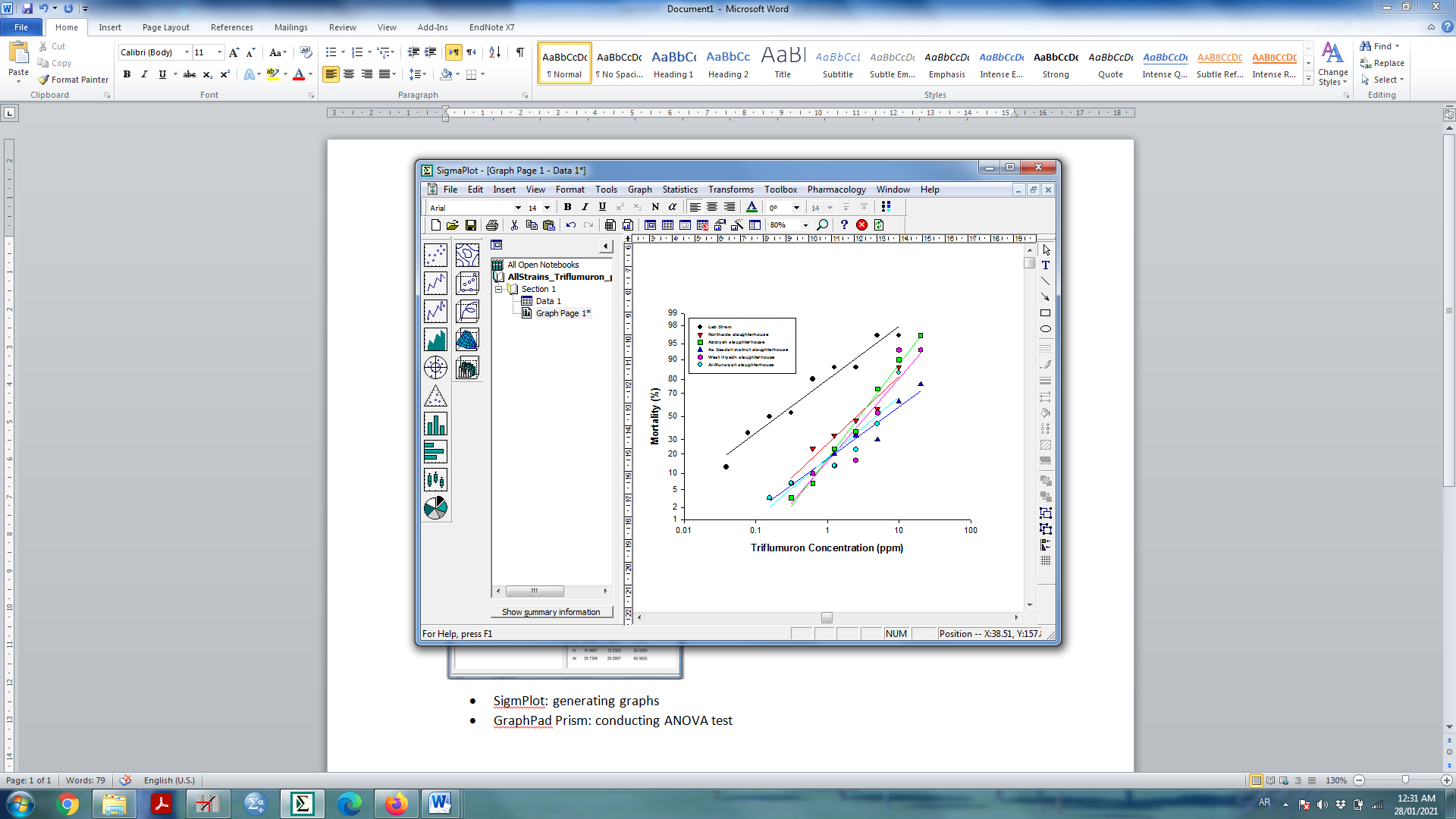


- GraphPad Prism (<https://www.graphpad.com/scientific-software/prism/>): conducting ANOVA test


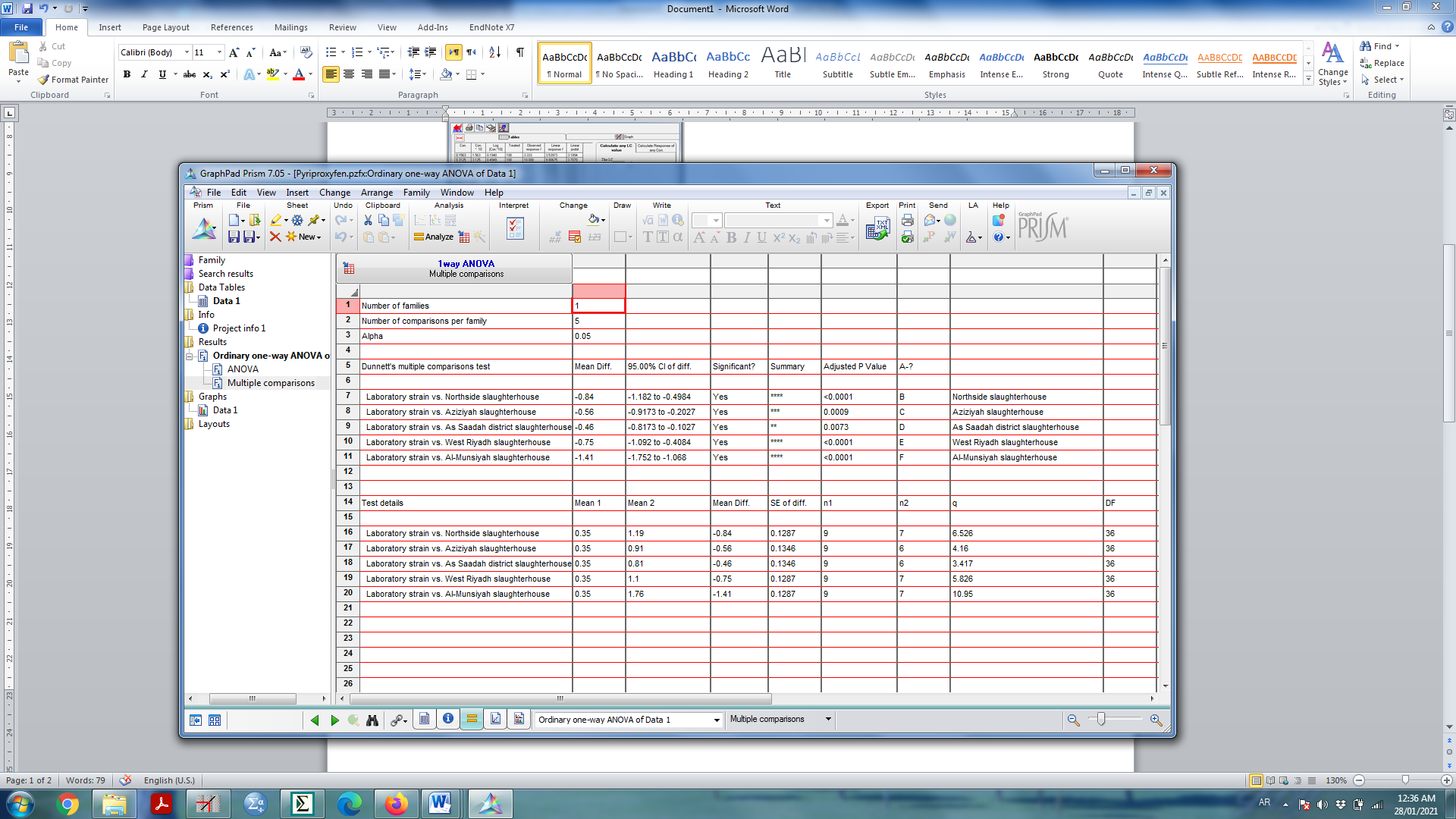


File extensions:

| File extension | sofware |
| --- | --- |
| .xls | Microsoft Excel |
| .ldp | LdP Line |
| .JNB | SigmPlot |
| .pzfx | GraphPad Prism |
