# Supplementary figures and images for: Evaluation of triflumuron and pyriproxyfen as alternative candidates to control house fly, Musca domestica L. (Diptera: Muscidae), in Riyadh city, Saudi Arabia
Source: PLoS One. 2021 Apr 8;16(4):e0249496. doi: 10.1371/journal.pone.0249496 (PMC8031380; doi:10.1371/journal.pone.0249496)

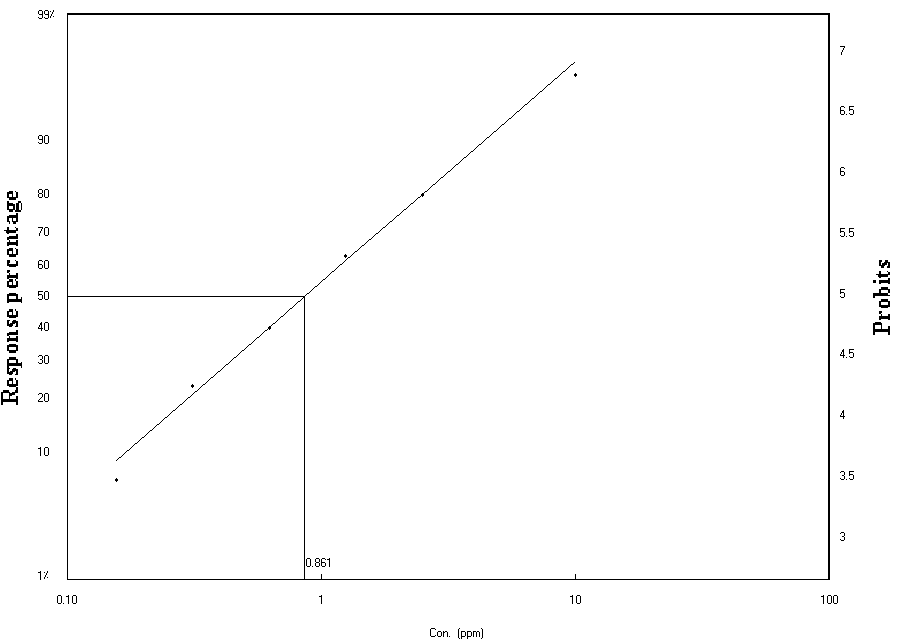

Supplement: S3 File — (ZIP) [file pone.0249496.s003.zip › S3_File/outputs/Pyriproxyfen/ldp_line_inputs_pyr_As Saadah.bmp]

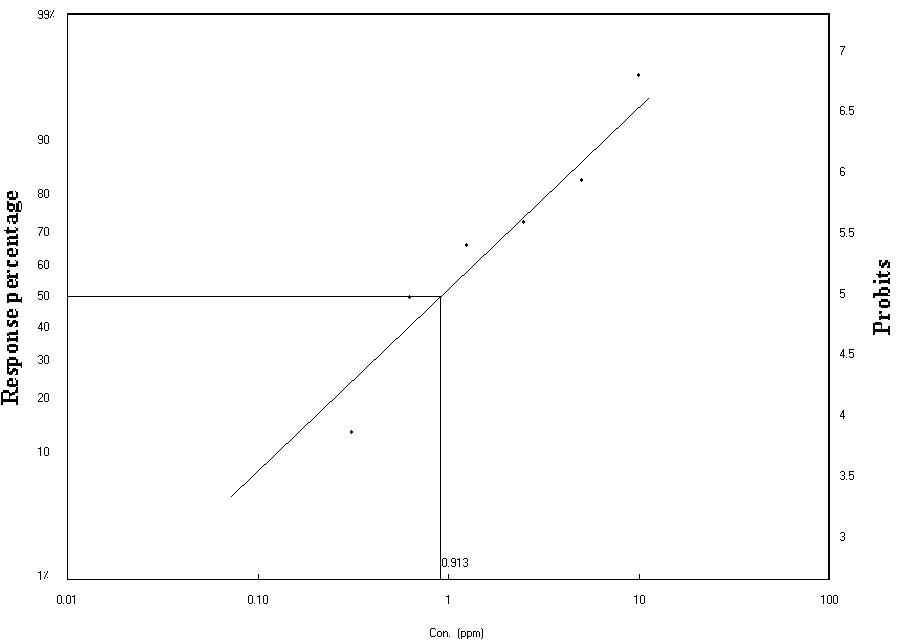

Supplement: S3 File — (ZIP) [file pone.0249496.s003.zip › S3_File/outputs/Pyriproxyfen/ldp_line_inputs_pyr_Aziziyah.bmp]

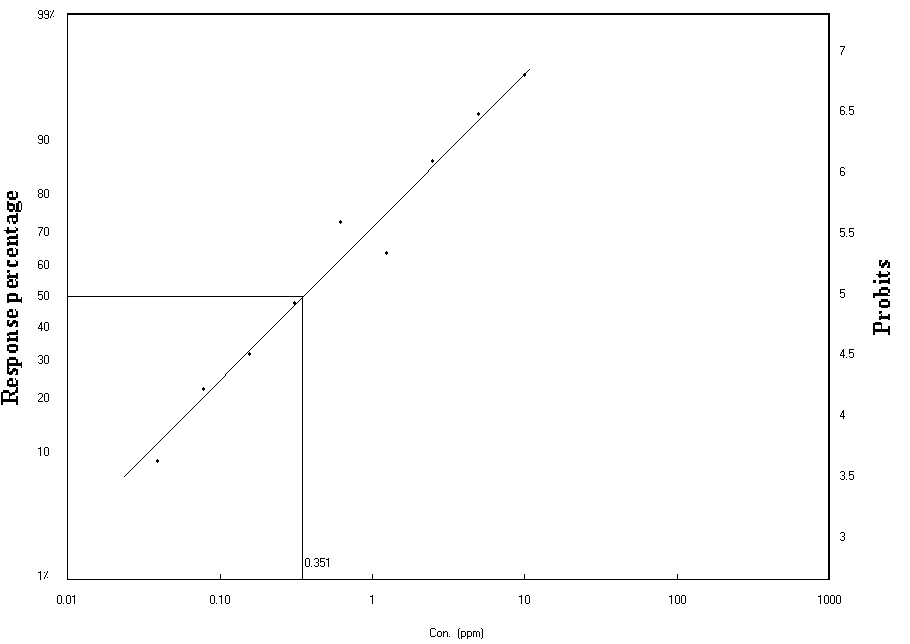

Supplement: S3 File — (ZIP) [file pone.0249496.s003.zip › S3_File/outputs/Pyriproxyfen/ldp_line_inputs_pyr_lab.bmp]

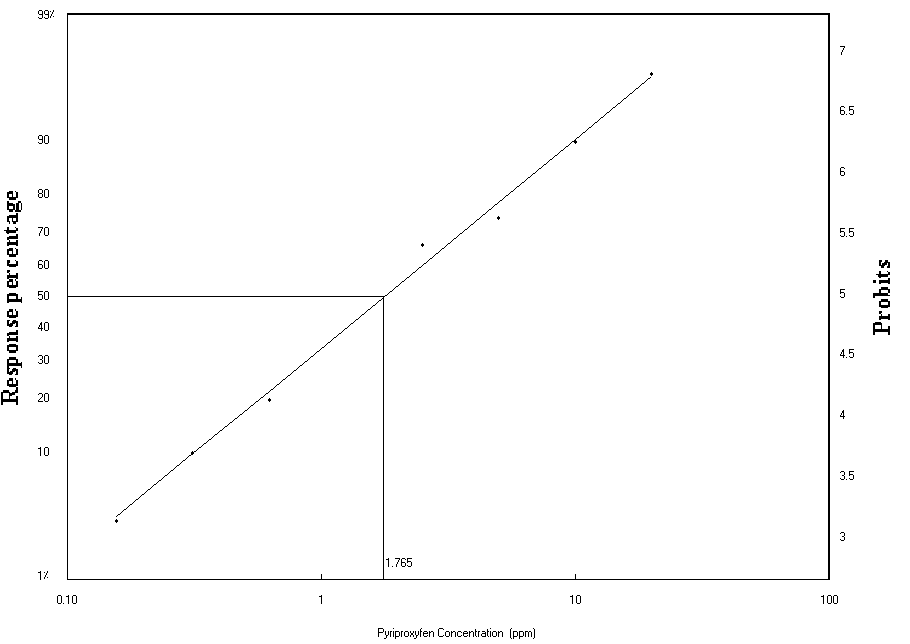

Supplement: S3 File — (ZIP) [file pone.0249496.s003.zip › S3_File/outputs/Pyriproxyfen/ldp_line_inputs_pyr_Munsiyah.bmp]

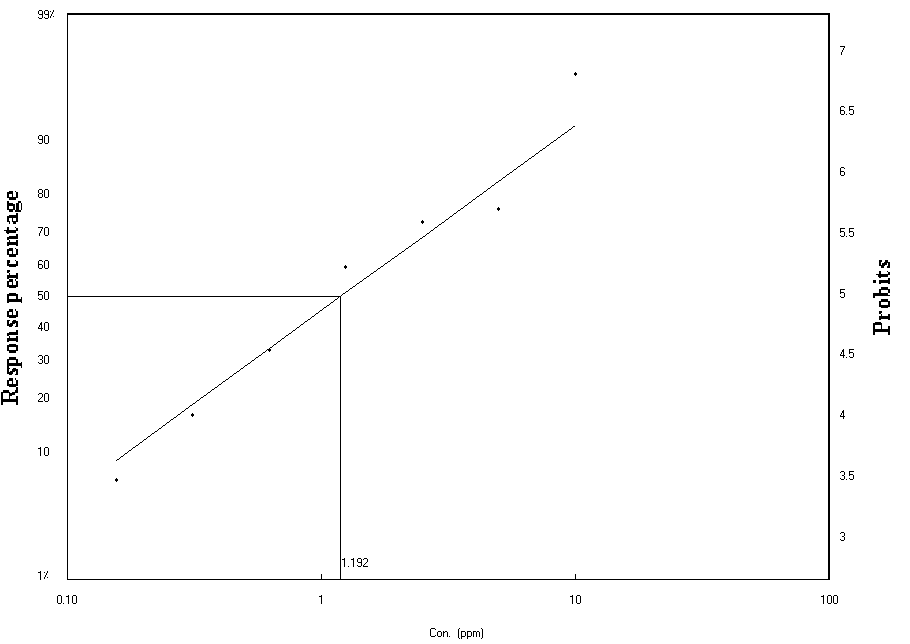

Supplement: S3 File — (ZIP) [file pone.0249496.s003.zip › S3_File/outputs/Pyriproxyfen/ldp_line_inputs_pyr_northside.bmp]

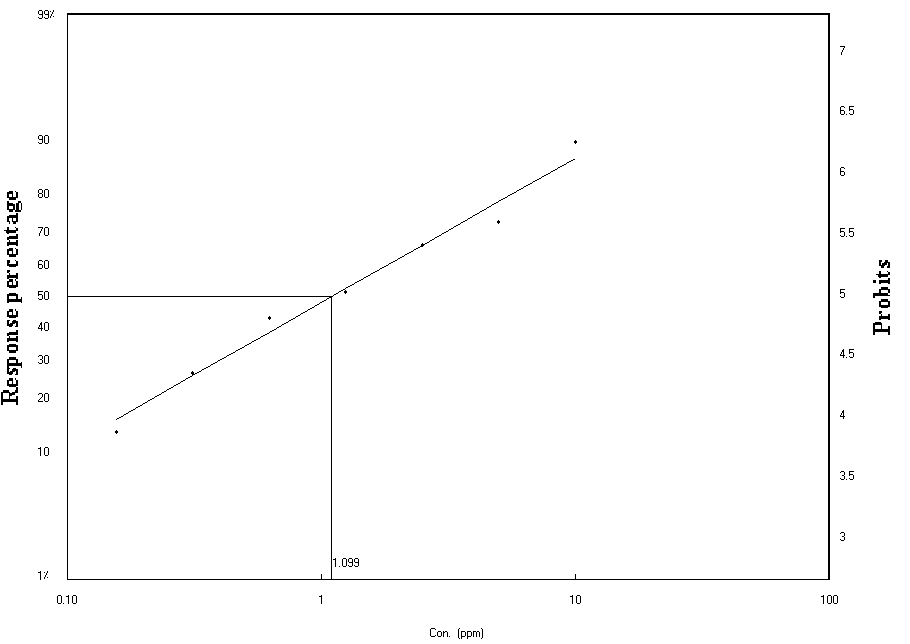

Supplement: S3 File — (ZIP) [file pone.0249496.s003.zip › S3_File/outputs/Pyriproxyfen/ldp_line_inputs_pyr_west.bmp]

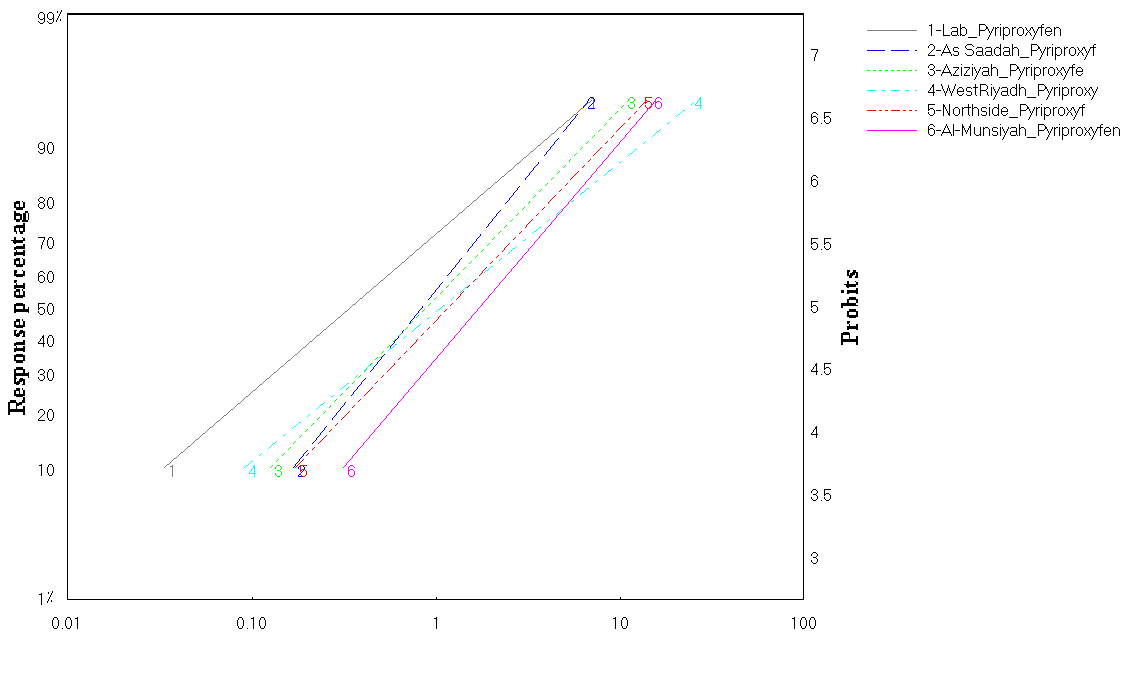

Supplement: S3 File — (ZIP) [file pone.0249496.s003.zip › S3_File/outputs/pyriproxyfen_compare.bmp]

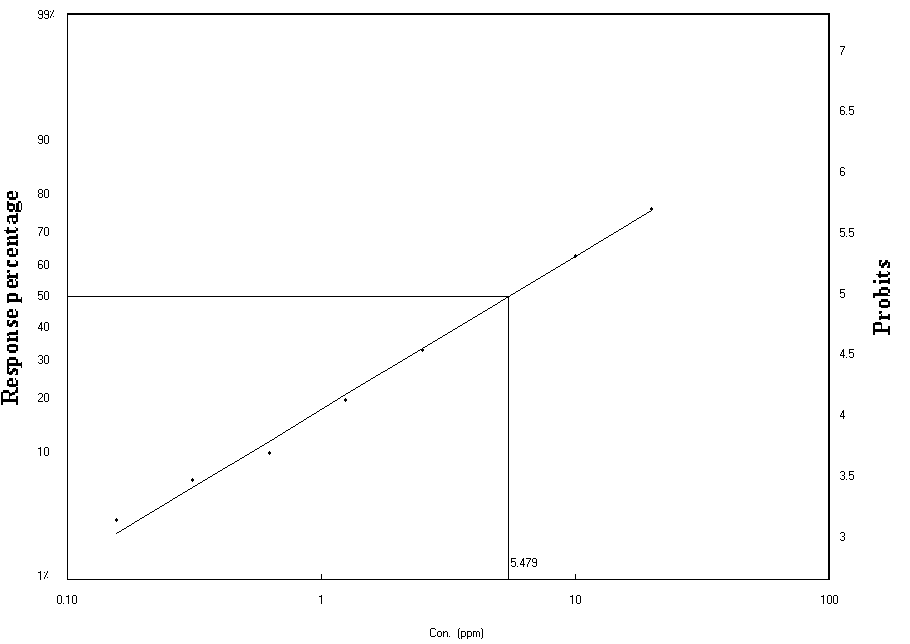

Supplement: S3 File — (ZIP) [file pone.0249496.s003.zip › S3_File/outputs/Triflumuron/ldp_line_inputs_tri_As Saadah.bmp]

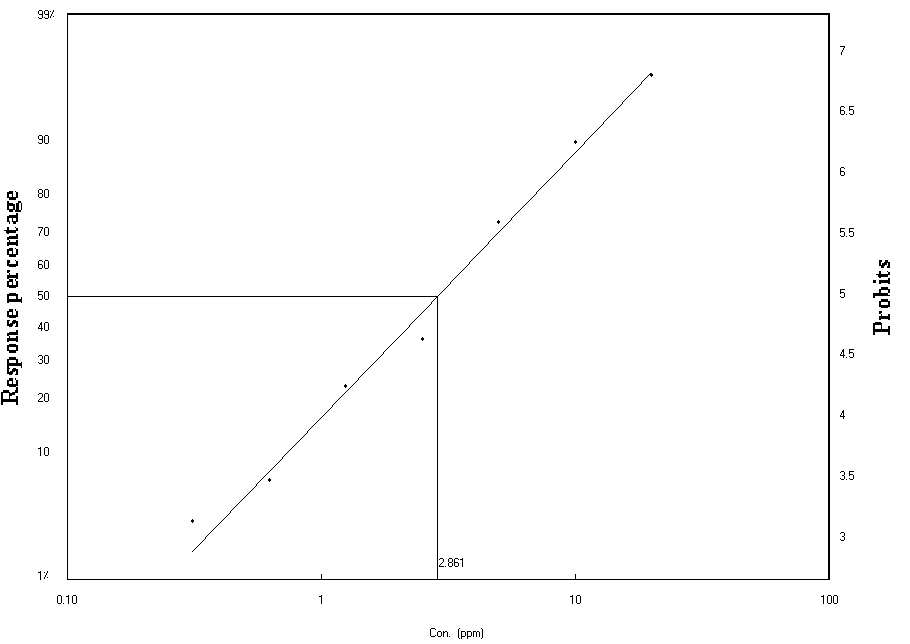

Supplement: S3 File — (ZIP) [file pone.0249496.s003.zip › S3_File/outputs/Triflumuron/ldp_line_inputs_tri_Aziziyah.bmp]

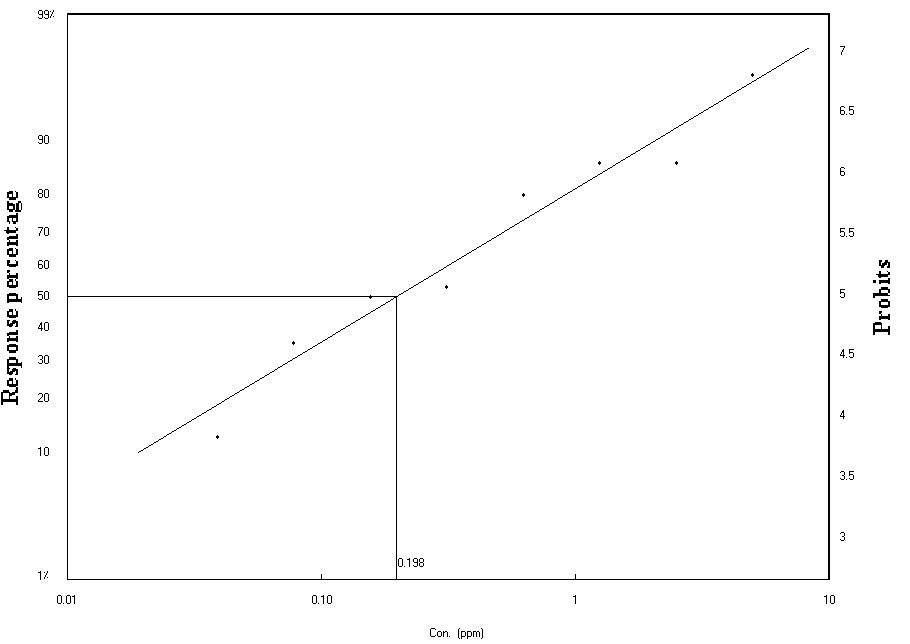

Supplement: S3 File — (ZIP) [file pone.0249496.s003.zip › S3_File/outputs/Triflumuron/ldp_line_inputs_tri_lab.bmp]

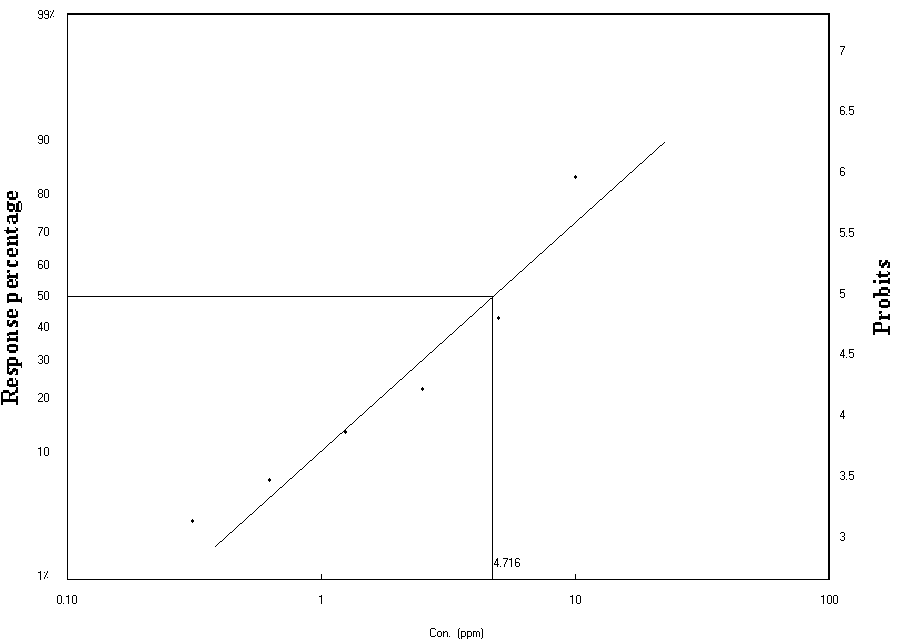

Supplement: S3 File — (ZIP) [file pone.0249496.s003.zip › S3_File/outputs/Triflumuron/ldp_line_inputs_tri_Munsiyah.bmp]

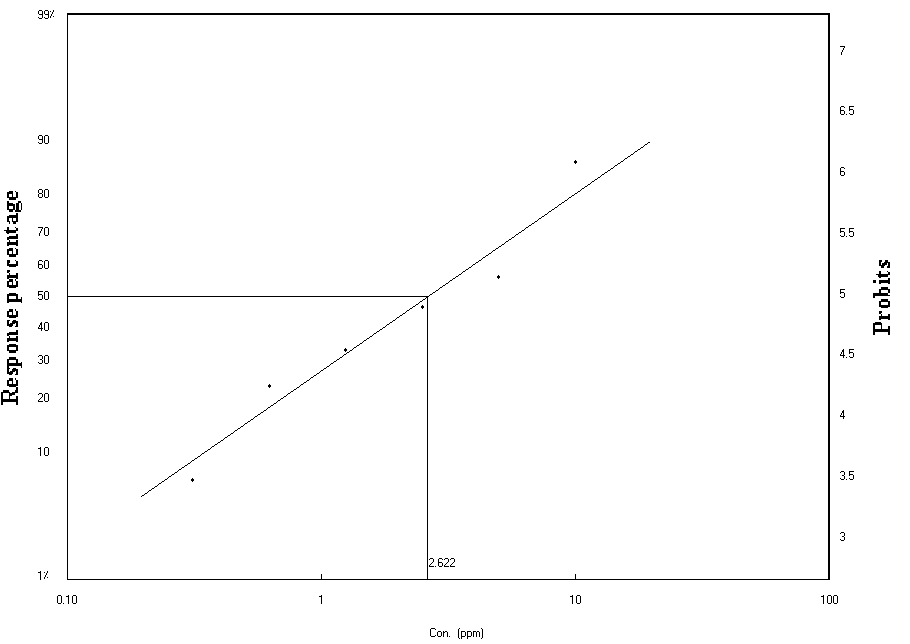

Supplement: S3 File — (ZIP) [file pone.0249496.s003.zip › S3_File/outputs/Triflumuron/ldp_line_inputs_tri_northside.bmp]

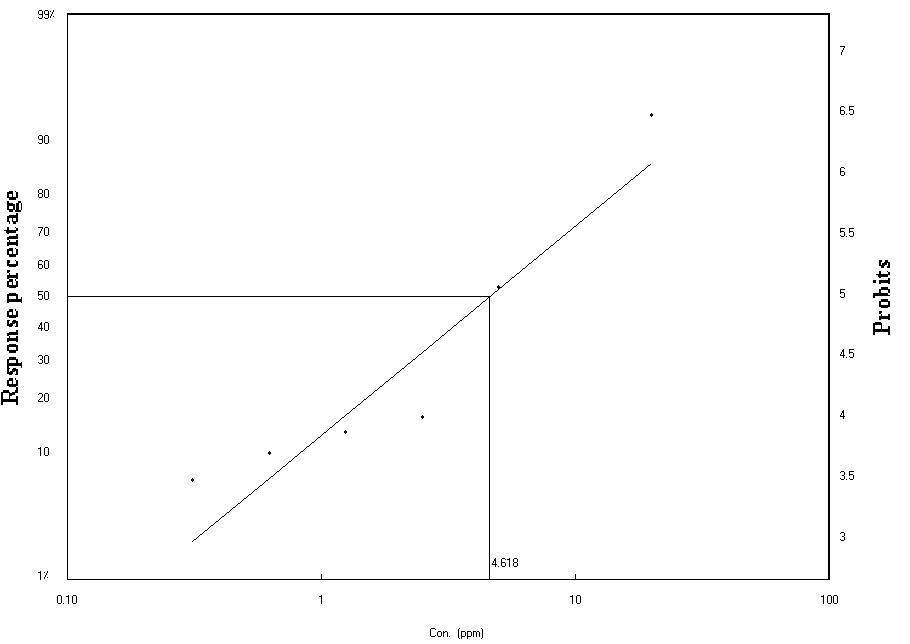

Supplement: S3 File — (ZIP) [file pone.0249496.s003.zip › S3_File/outputs/Triflumuron/ldp_line_inputs_tri_west.bmp]

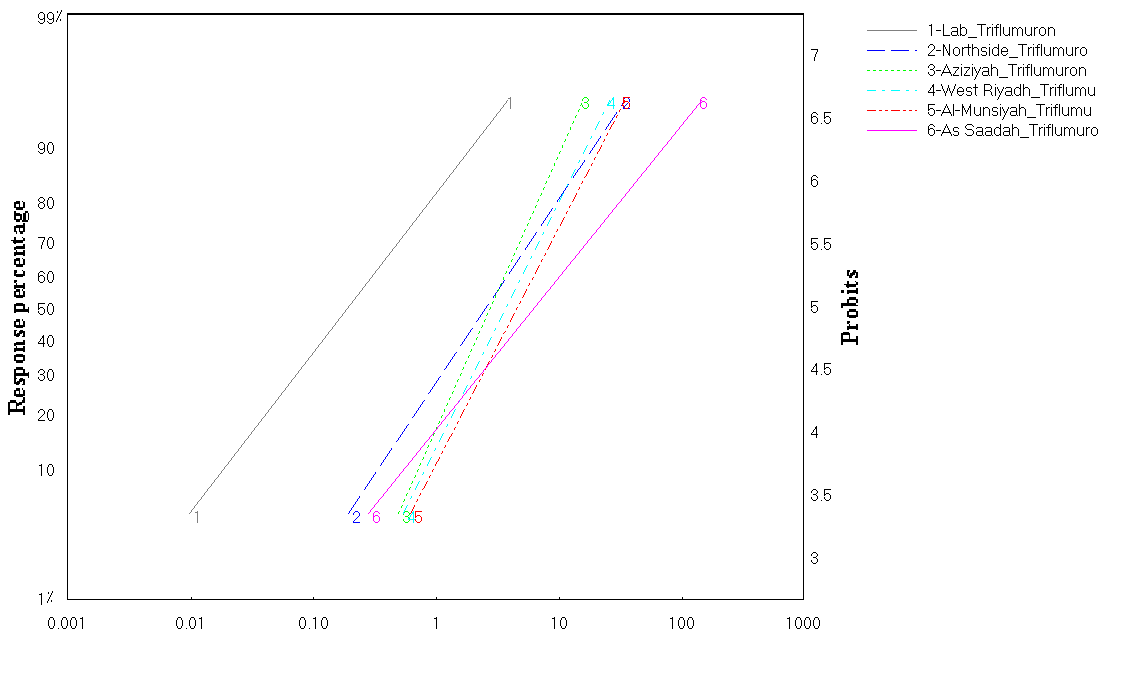

Supplement: S3 File — (ZIP) [file pone.0249496.s003.zip › S3_File/outputs/triflumuron_compare.bmp]

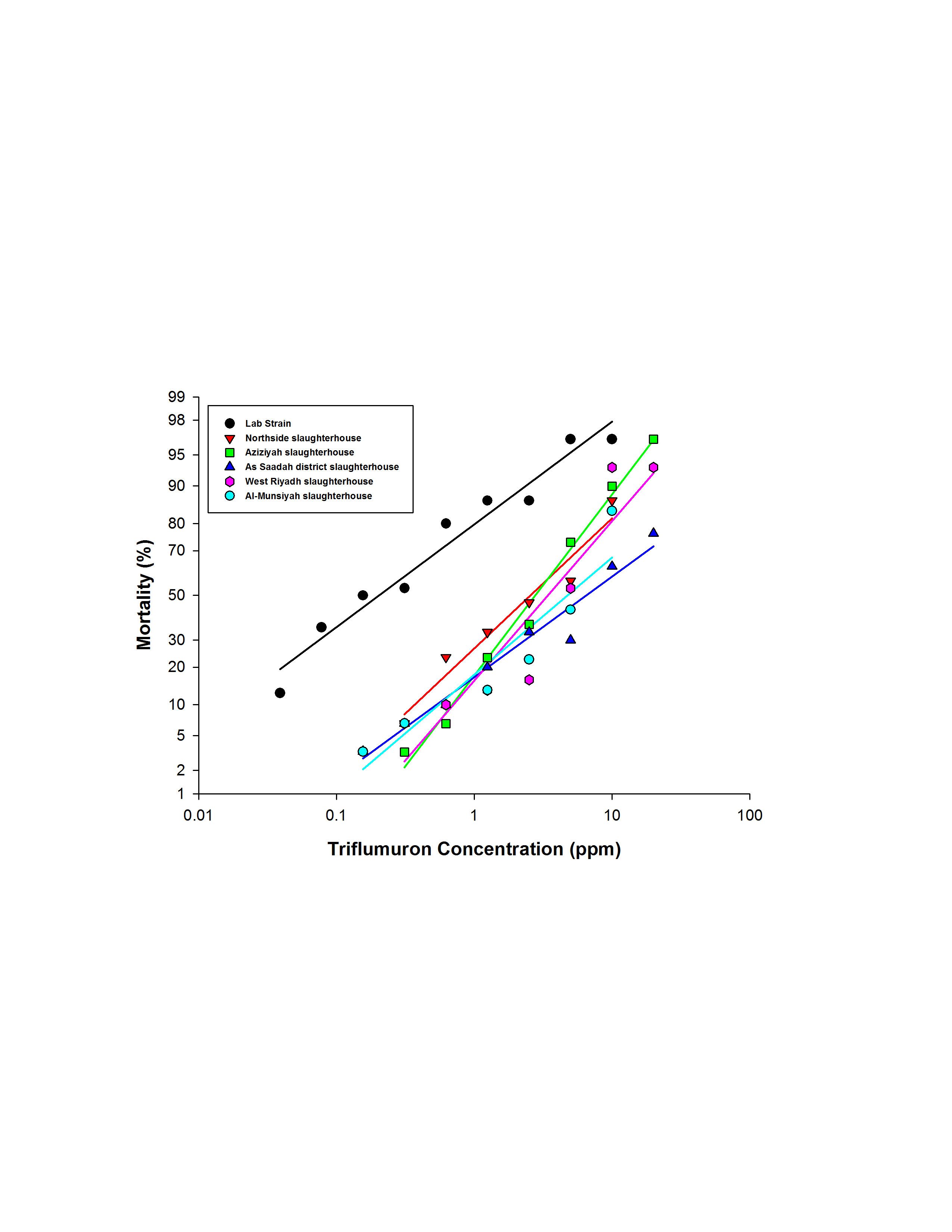

Supplement: S4 File — (ZIP) [file pone.0249496.s004.zip › S4_File/Fig 1.TIF]

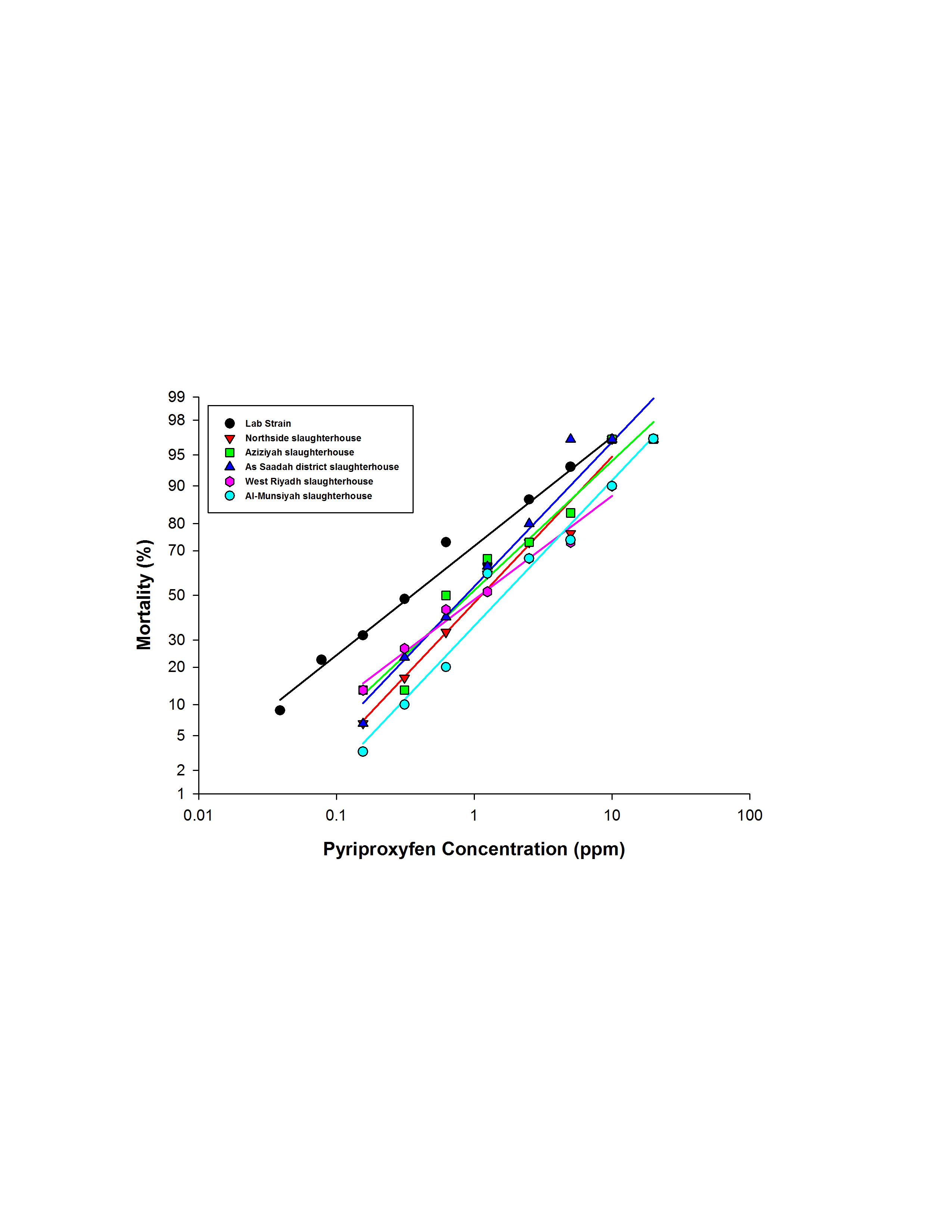

Supplement: S4 File — (ZIP) [file pone.0249496.s004.zip › S4_File/Fig 2.TIF]
